# Exploring the relationship between exanthematous diseases and early-onset type 1 diabetes in children

**DOI:** 10.1016/j.jped.2025.03.001

**Published:** 2025-03-11

**Authors:** Massimo Pettoello-Mantovani, Pietro Ferrara, Ida Giardino

**Affiliations:** a Union of National European Pediatric Societies and Associations, European Pediatric Association, Berlin, Germany; b Italian Academy of Pediatrics, Milan, Italy; c Department of Clinical and Experimental Medicine, University of Foggia, Foggia, Italy

## Introduction

The rising global incidence of Type 1 diabetes mellitus (T1DM) calls for innovative research to further explore its pathophysiological mechanisms, improve early detection, and refine treatment strategies. The study on the association between exanthematous diseases and early age at Type 1 diabetes diagnosis, conducted on a Brazilian cohort by Lopes et al.,^1^ offers compelling insights into the potential role of viral and bacterial exanthematous diseases in shaping the age of T1DM onset in children. This retrospective study conducted in Brazil provides an intriguing examination of how childhood infections, including rubella, measles, mumps, and scarlet fever, might correlate with an earlier age of T1DM diagnosis. Additionally, it sheds light on the influence of socioeconomic status on the timing of T1DM onset. While the study presents a unique contribution to the understanding of T1DM epidemiology in Brazil, its findings also offer broader implications for global public health research.

## Main insights from the Brazilian study and their implications

The study on the Brazilian children cohort,^1^ identifies a clear association between early T1DM diagnosis and a history of childhood infections such as rubella, measles, and mumps.^2^, ^3^, ^4^, ^5^ Specifically, children with a history of these exanthematous diseases were diagnosed with T1DM at a significantly younger age compared to those without such a history. This finding aligns with earlier studies suggesting that viral infections, particularly those that occur during childhood, may act as environmental triggers for autoimmune processes that lead to the destruction of pancreatic beta cells.

For instance, the study's finding that individuals with a history of rubella, measles, or mumps had a 35% to 40% lower age at T1DM diagnosis reinforces the hypothesis that infections during childhood could accelerate the autoimmune process. These findings mirror earlier research, including Italian and Finnish studies, which observed an association between these specific childhood infections and T1DM risk.^6^, ^7^, ^8^ While the exact mechanisms remain unclear, it is plausible that these viral infections may disrupt immune regulation, triggering an autoimmune response that targets the pancreatic beta cells. One particularly thought-provoking element of the study is its exploration of socioeconomic status. The study reveals that children from non-high socioeconomic classes tend to experience an earlier onset of T1DM. This finding highlights the broader social determinants of health that contribute to the timing of disease diagnosis. Children from lower socioeconomic backgrounds may face barriers to accessing early diagnosis and care, which could lead to delayed interventions and more severe disease progression. The influence of socioeconomic factors on T1DM onset also raises important questions about healthcare equity and the need for improved access to preventive care and early diagnosis, particularly in underserved populations.

## The role of exanthematous diseases in the pathogenesis of T1DM

Type 1 diabetes, characterized by the destruction of insulin-producing beta cells in the pancreas, has been linked to both genetic predispositions and environmental factors.^9^ Historically, the autoimmune destruction associated with T1DM has been attributed to various triggers, including viral infections.^9^^,^^10^ The research presented by the Brazilian group^1^ aligns with previous studies investigating the potential role of infections in T1DM pathogenesis, offering a novel perspective by focusing on the intersection of early-life exanthematous diseases and T1DM diagnosis age. To this regard, the Brazilian context is particularly significant for this study. As the incidence of T1DM continues to rise globally,^11^ including in Latin America, understanding local factors that might contribute to earlier diagnoses of T1DM is crucial. The investigation by Lopes et al. into the relationship between common childhood infections and T1DM onset in Brazilian children could provide valuable insights for clinicians and public health. The study's design, which is based on a retrospective cohort analysis of 596 patients diagnosed with T1DM between 1981 and 2023, leverages a comprehensive sample that spans over four decades. This extensive timeframe is a major strength, allowing for the exploration of trends in disease onset across different time periods, socioeconomic strata, and healthcare systems. By including both private and public healthcare patients, the study captures a broad and representative demographic, enhancing its external validity.

One of the study's standout features is the standardized approach to data collection. Most importantly, the same researcher managed patient assessments throughout the study period, which minimizes inter-observer variability and ensures consistency in diagnoses and medical histories. Although the retrospective nature of the study, relying on medical records and patient self-reports, is a limitation, the thoroughness of the data collection process, which was combined with regular reassessments during follow-up consultations, helped mitigating some of the potential biases inherent in such designs.

## Viral infections and their implications in the earlier onset of T1DM

The association between viral infections and T1DM onset is unclear. However, this study contributes valuable context by emphasizing the role of specific viral diseases in childhood. The immune mechanisms behind this association are still being investigated, but several theories exist. One prominent hypothesis is that viral infections may alter the expression of genes within the HLA class, thereby modulating immune responses and increasing susceptibility to autoimmune disease. In this way, viruses may initiate the process by which the immune system mistakenly attacks and destroys the insulin-producing beta cells.^12^ Moreover, the study touches on the possibility of a dual role for infections. Some viruses may trigger T1DM, while others may confer protection. For example, a French study mentioned by the authors in the introduction found an inverse correlation between varicella (chickenpox) and T1DM risk.^13^ This paradox suggests that while some infections may hasten the onset of T1DM, others may offer a form of immune modulation that helps prevent it. The ongoing exploration of viral infections as both risk factors and potential protective agents underscores the complexity of T1DM's pathogenesis and the need for further research.^14^

## The role of socioeconomic factors

The study's findings^1^ regarding socioeconomic status are also noteworthy. It is well-established that individuals from lower socioeconomic backgrounds often face greater health challenges, including higher exposure to environmental and social risk factors, limited access to healthcare, and delayed diagnosis.^13^ The association between lower socioeconomic status and earlier T1DM diagnosis is likely multifactorial.^13^^,^^15^ On the one hand, the study points to a lack of access to timely medical care as a potential factor. On the other hand, socioeconomic disparities in health outcomes, including the management of chronic diseases like T1DM, have been widely documented in the literature.^15^^,^^16^

The study confirms that in Brazil, as well as in the rest of the world, socioeconomic status plays a significant role in determining access to health services and educational resources. Children from wealthier families are more likely to have access to regular health screenings and preventive care, potentially leading to earlier diagnosis and better management of chronic conditions. Conversely, children from lower-income families may face barriers to early diagnosis, leading to more severe complications and potentially contributing to an earlier onset of disease.

### Future research

Although the study by Lopes et al.^1^ has some intrinsic limitations, it provides valuable insights and suggestions for future research. The reliance on patient self-reports regarding the history of exanthematous diseases may introduce recall bias, particularly when patients or their parents struggle to accurately recall past infections. Additionally, the lack of data on vaccination status is a notable limitation, as vaccination plays a key role in preventing many infections linked to the onset of T1DM. However, the Brazilian study notably paves the way for future research that, by including vaccination data, would be invaluable in further clarifying the complex relationship between immunization and autoimmune diseases like T1DM.

Furthermore, a longitudinal cohort study, following children over time and assessing the development of T1DM in relation to their exposure to infections, would provide more definitive evidence of causality. Finally, long-term, prospective studies with larger, more diverse populations would also help to confirm the generalizability of these findings and refine our understanding of how specific exanthematous diseases influence T1DM pathogenesis.

## Conclusion

The Brazilian cohort study on the link between exanthematous diseases and early-onset Type 1 Diabetes^1^, provides valuable new insights into the role of childhood infections in the early onset of Type 1 diabetes. The associations between rubella, measles, mumps, and lower socioeconomic status with earlier T1DM diagnoses suggest that both viral exposures and social determinants of health play critical roles in shaping the course of this disease.^17^^,^^18^ The findings are promising, and suggest that more research will be useful to unravel the complex immune mechanisms behind these associations and to explore potential preventive strategies.

As the global incidence of T1DM continues to rise, studies like this one offer a critical opportunity to understand the environmental triggers that contribute to its development.^19^ By expanding our knowledge of the factors influencing T1DM onset, researchers and healthcare professionals can work toward more effective prevention, earlier detection, and improved management strategies for those affected by this challenging disease.^20^

## Conflicts of interest

The authors declare no conflict of interest.
